# Filling the Gaps for Enhancing the Effectiveness of Community-Based Programs Combining Treatment and Prevention of Child Malnutrition: Results from the Rainbow Project 2015–17 in Zambia

**DOI:** 10.3390/ijerph15091807

**Published:** 2018-08-22

**Authors:** Stefania Moramarco, Giulia Amerio, Jean Kasengele Chipoma, Karin Nielsen-Saines, Leonardo Palombi, Ersilia Buonomo

**Affiliations:** 1 Department of Biomedicine and Prevention, University of Rome Tor Vergata, via Montpellier, Rome 00133, Italy; stefania.moramarco@gmail.com (S.M.); palombi@uniroma2.it (L.P.); ersilia.buonomo@uniroma2.it (E.B.); 2 Rainbow Project Association Pope John 23rd, 5656 Chinika Road, Ndola 10101, Zambia; giulia.amerio@gmail.com; 3 Ndola District Health Office, 1307 Naidu Close, Ndola 10101, Zambia; jeankasengele@gmail.com; 4 Department of Pediatrics, David Geffen UCLA School of Medicine School of Medicine, Los Angeles, CA 90095, USA

**Keywords:** child malnutrition, community-based management of acute malnutrition—CMAM, moderate acute malnutrition—MAM, severe acute malnutrition—SAM, supplementary feeding programs—SFP, underweight, Zambia

## Abstract

Background: Child malnutrition, in all its forms, is a public health priority in Zambia. After implementations based on a previous evaluation in 2012–14 were made, the efficacy of the Rainbow Project Supplementary Feeding Programs (SFPs) for the integrated management of severe acute malnutrition (SAM), moderate acute malnutrition (MAM), and underweight was reassessed in 2015–17. Methods: The outcomes were compared with International Standards and with those of 2012–14. Cox proportional risk regression analysis was performed to identify predictors of mortality and defaulting. Results: The data for 900 under-five year-old malnourished children were analyzed. Rainbow’s 2015–17 outcomes met International Standards, for total and also when stratified for different type of malnutrition. A better performance than 2012–14 was noted in the main areas previously identified as critical: mortality rates were halved (5.6% vs. 3.1%, *p* = 0.01); significant improvements in average weight gain and mean length of stay were registered for recovered children (*p* < 0.001). HIV infection (5.5; 1.9–15.9), WAZ <–3 (4.6; 1.3–16.1), and kwashiorkor (3.5; 1.2–9.5) remained the major predictors of mortality. Secondly, training community volunteers consistently increased the awareness of a child’s HIV status (+30%; *p* < 0.001). Conclusion: Rainbow SFPs provide an integrated community-based approach for the treatment and prevention of child malnutrition in Zambia, with its effectiveness significantly enhanced after the gaps in activities were filled.

## 1. Introduction

Childhood malnutrition remains a major public health problem throughout the developing world, being the underlying factor for nearly half of all yearly under-5 deaths from preventable causes [1]. It is estimated that more than 50 million children worldwide are affected by acute malnutrition, with 16 million having Severe Acute Malnutrition (SAM) and a further 33 million having Moderate Acute Malnutrition (MAM) [2]. Acute malnutrition, if untreated, is an attributable cause of death; 12.6% of the 6.9 million deaths worldwide among children under five years of age are due to acute malnutrition [3]. The community-based management of acute malnutrition (CMAM) is unequivocally advocated as an effective program to address acute malnutrition in children aged 6 to 59 months and it is globally implemented in 55 countries. This approach, which provides services to local communities by decentralized treatment points within existing healthcare facilities, is widely used across multiple humanitarian agencies both for SAM and MAM [4,5,6]. Within the CMAM, targeted Supplementary Feeding Programs (SFPs) are recognized as best practice for treating MAM and preventing the deterioration into SAM in the emergency nutrition response [7]. However, more evidence-based research is needed to evaluate the effectiveness of a combined approach integrating preventive and curative interventions [8], adapted to different scenarios and non-emergency settings [6].

In Zambia, malnutrition remains one of the most serious problems among children under five years of age. This condition is estimated to underlie 52% of all under-five deaths [9] (64 per 1000 live births in 2015) [10]. In 2015, the wasting prevalence in Zambia was 6.3% [11], which was off course from the World Health Assembly Nutrition target to “reduce and maintain childhood wasting to less than 5% by 2025” [12]. The picture of malnutrition has been exacerbated by high rates of HIV/AIDS (UNAIDS estimates that 85,000 children below 14 years of age were HIV infected in 2015 [13]) and by an exponential rise in tuberculosis (TB) over the last 3 decades, with children accounting for <10% of incident TB cases annually [14], more than 50% of whom are HIV co-infected [15,16]. Traditional CMAM is not widely available in the country and most areas are not covered by nutrition-specific interventions targeting acute malnutrition [17]. However, the Zambian Government still remains committed to the scale-up provision of high impact services with a special focus on maternal and child health [18]. In the Ndola area, the Rainbow Project, under the Association Pope John 23rd, is the only locally well-implemented program with an integrated community-based approach for the management of child malnutrition that combines MAM/SAM/underweight treatment and prevention, operating through the SFPs since 1998 [19].

The aim of the current study was primarily to demonstrate the Rainbow SFPs’ effectiveness in the community-based management of child malnutrition in Zambia, by presenting an innovative approach run by well-trained community volunteers, integrating treatment and prevention of MAM, SAM, and being underweight. Therefore, program outcomes between the years of 2015–17 were evaluated and compared with International Standards and previous study results (Rainbow 2012–14), after filling the gaps in prior activities [20]. Secondly, the current study aimed to demonstrate that Rainbow SFPs can act as an integrated program for nutrition and child health promotion, coupling growth surveillance with HIV counseling and diagnosis, facilitating access to HIV/TB treatment and overall care for Zambian children.

## 2. Materials and Methods

### 2.1. Setting

The Rainbow Project operates 11 SFPs for Zambian malnourished children (ages 6–59 months) in the Ndola area (9 located in urban areas: Twapia, Nkwazi, Kabushi, Kaloko, Kawama, Chifubu, Pamodzi, Kanyala and Mackenzie; 2 located in rural areas: Baluba and Chikumbi) with a particular focus on community mobilization and capacity building activities. All of the centers are run by leaders of small NGOs and Community-based Organizations (CBOs), and are coordinated by professionals of the Rainbow Office, in close network with health facilities, local clinics running Outpatient Therapeutic Programs (OTP), the Children’s Hospital, the Ndola District Health Management Teams (DHMTs), and other local authorities. In CMAM programs, children with MAM are the main target of SFPs, while SAM rehabilitation is addressed by OTPs and/or Inpatient Care (IC) [21,22]. However, within the Zambian context, since the access to OTP/IC is restricted and/or the supply of ready-to-use therapeutic food (RUTF) is erratic in most areas in which Rainbow operates, children with SAM are enrolled in Rainbow SFPs, in addition to referral to OTP/IC as the best practice. This choice has been made for ethical and humanitarian reasons, in order to facilitate the access of children/families most in need to nutritional supplementation, health education and all the other activities that are part of the Rainbow approach. Children who are underweight are enrolled in the program with the viewpoint that optimizing healthy child growth and improving nutritional status can have an impact on reducing and preventing rates of wasting [23,24]. Therefore, Rainbow SFPs provide an effective integrated approach for child malnutrition management, combining treatment and the prevention of MAM/SAM/and being underweight.

### 2.2. Rainbow SFPs Activities

Rainbow SFPs’ protocol includes both nutrition-specific or direct interventions (growth monitoring and supplementary food) and nutrition-sensitive or indirect interventions (nutritional counseling, health skills for guardians, and child health promotion). Nutrition-specific activities (anthropometric assessment, on-site feeding, cooking demonstrations, food handouts: local food—maize flour, groundnuts, sugar, oil—and a packaged fortified blended flour—high energy protein supplement/HEPS made with maize, soya and enriched with vitamins and minerals), coupled by nutrition-sensitive interventions (nutritional counseling and health education) are performed on a weekly basis. The food schedule was recently revised in order to improve the quantity and quality of high energy protein supplement/HEPS from the previous rations of 2012–14: the amount of HEPS distributed was redoubled for providing a daily ration of 150 g; and guardians/mothers were counseled to add sugar and oil when cooking it [23,25]. In Rainbow’s SFPs, all the children receive the same food supplements, regardless of the type of malnutrition and/or simultaneous admission in OTP.

All community volunteers/operators are appropriately trained and constantly updated in Infant and Young Child Feeding (IYCF) practices promoted by the Zambian Government. 

Supporting HIV voluntary counseling and testing (VCT) and active HIV case-finding are also part of the SFP routine activities. Guardians/mothers of children with an unknown HIV status at the time of admission are counseled and encouraged to go to the nearest health facility for an HIV test for both mother and child. Personnel are trained in confidentiality issues (e.g., counseling in the context of prevention of mother-to-child transmission PMTCT), with specific refresher courses attended within the last years.

Home visits performed by community volunteers during nutritional rehabilitation are necessary to encourage good adherence to treatment and to tackle determinants of malnutrition at the household level.

Revision of the food schedule (doubling HEPS quantity and improving quality) and staff training support in HIV counseling and testing were the two main activities implemented during the last two years, after critical areas for improvement were identified during the previous program evaluation in 2012–14 [20]. Nutritional counseling, emphasizing the use of locally available food, was also integrated as a routine activity of the SFPs, with pilot study results discussed elsewhere [26].

### 2.3. Study Population and Data Collection 

Data on Zambian malnourished children followed from July 2015 to April 2017 in the Rainbow Project SFPs were analyzed using a community-based retrospective observational cohort study design. General pediatric information and socio-demographic characteristics, health and nutritional parameters were entered in a register created ad hoc for the project. General information included the date of birth, age, gender, and sibling count. Socio-demographic data recorded included the family history (parents’ marital status, relationship, and age of the guardian) and housing information (area of stay, address, household conditions). The health information included disability, medical complications and/or illness, enrollment in OTP, and HIV status. The latter (HIV status), was ascertained from the prevention mother-to-child transmission (PMTCT) section of the child under-five card released from the primary health care facilities of the Zambia Health Ministry. A color code was assigned to sensitive information (such as HIV status). Nutritional parameters included anthropometric measurement (weight, MUAC, edema). The data were registered after verbal consent of caregivers and in full respect of confidentiality and they were then collected from different sites and entered into a database with the removal of personal identifiers.

### 2.4. Anthropometric Assessment and Malnutrition Classification 

Malnourished children enrolled in Rainbow SFPs were recruited through community outreaches or referred from local health facilities. The anthropometric assessment consisted in measuring weight, Mid-Upper Arm Circumference (MUAC), and checking for bilateral pitting edema. Children were assessed without clothes or footwear; their weight (in kilograms) was measured using a mechanical baby scale graduated by 0.1 kg increments (salter 235). MUAC (in centimeters) was measured using a simple colored plastic strip (standardized UNICEF tape). Bilateral pitting edema was checked by applying gentle thumb pressure on the dorsum of the feet and assessing for residual depression; edema was detected as different grades.

The children were admitted to SFPs by using a two-priority criteria system of enrollment: first priority was given to acute malnutrition (SAM or MAM) and second priority to underweight status. Definition of SAM (MUAC ≤ 11.5 cm, and/or edema) and MAM (MUAC > 11.5 and ≤12.5) was made according to the WHO/UNICEF criteria [27] and the Integrating Management of Acute Malnutrition (IMAM) guidelines of the Zambian Ministry of Health [28] for children aged 6 to 59 months. As recently indicated in the updated WHO guidelines, in order to achieve the early community identification of malnourished children, Rainbow community volunteers measured the MUAC and examined children for pitting edema, reserving the assessment of weight for height and weight for length (WHZ/WLZ) within primary health care facilities and hospitals [29]. Underweight was defined as a weight-for-age Z-score (WAZ) <–2 [30].

If a child qualified at the same time for different criteria, the enrollment in SFPs was made considering the most severe condition of malnutrition. All children with MAM or being underweight without health complications were enrolled directly, while when reporting health complications they were first referred to the nearest health facility for medical care. If the child was found to have SAM without medical complications (either marasmus or kwashiorkor grade 1 and 2), he/she was primarily enrolled in OTP following the best practice guidelines [28]. Simultaneously those children were enrolled in SFP for ethical reasons, as previously reported. If the health staff identified a child as having SAM with medical complications, he/she was referred to the Arthur Davidson Children’s Hospital for IC and then returned to Rainbow SFP when discharged from the IC. All children stayed in the program until the SFP discharge criteria were met: for two consecutive weeks the MUAC should be >12.5 cm, the edema should be absent, or there should be a 15% weight gain to be considered if being underweight was the admission criteria [23].

### 2.5. Program Outcomes and Performance Indicators

Standard outcomes included recovery rate, death rate, and default rate. Recovered/cured was defined as an individual who met the discharge criteria. Defaulter was defined as a child lost to follow up for three consecutive weeks. A child was classified as a “defaulter” when he/she dropped out of the study due to refusal or it was not possible to locate the child and make a home assessment. Death was registered when occurring during the time the patient was enrolled in the program. Early mortality and defaulting (within 15 days from enrollment) were excluded because they might not be directly attributable to the performance of the SFPs. Individuals who did not complete their rehabilitation because they moved to another area were considered transferred; this outcome was not included in the performance evaluation because of the current absence of published targets. The length of stay and weight gain were considered additional indicators for targeted SFPs. The mean length of stay expressed the time of stay for recovered children; the mean weight gain expressed the average number of grams gained per kilograms per day among children who were cured. For humanitarian and ethical reasons, nutritional treatment was provided until children reached the recovery goals (treat-to-goal), so none were categorized as non-cured/non-responder (defined as cases that did not reach discharge criteria after a pre-defined length of time). The program outcomes were compared with exit categories for targeted SFPs from the Sphere Project (recovery, death, and defaulter rate) and UNHCR guidelines (mean length of stay, and average weight gain). The Sphere Standards are the typical criteria used for assessing the effectiveness of SFP [31]. The UNHCR guidelines are intended as a practical guide to design, implement, monitor, and evaluate selective feeding programs in emergency situations [23]. In addition, outcomes of Rainbow 2015–17 were compared with those previously published in 2012–14, in order to evaluate the impact of adjustments on the program performance.

### 2.6. Statistical Analysis 

The data were extracted from the Rainbow database and analyzed using the SPSS software system 21.0 (IBM, Somers, NY, USA). Weight-for-age Z-scores (WAZ) and MUAC-for-age Z-scores (ZMUAC) were calculated using the WHO Anthro Software (Version 3.2.2, January 2011, WHO, Geneva, Switzerland) [32]. Descriptive data and variables measured were presented as means with standard deviations (SD). The Odds ratios 95% Confidence Intervals (OR; 95% CI) were calculated between age and general acute malnutrition, and between different types of severe acute malnutrition (marasmus and kwashiorkor) and length of stay. A descriptive analysis was performed for the entire study population to estimate the proportion of children who recovered, died, and defaulted during the intervention phase. Rainbow 2015–17 outcomes were compared with those of Rainbow 2012–14, with student *t*-tests for assessing the statistical significance of differences between continuous variables and Z-test for independent proportions. Univariate and multivariate Cox regressions were performed to identify the main predictors of mortality and defaulting (hazard ratio: HR, 95% CI). Because there were multiple independent variables, a stepwise forward regression approach was used.

## 3. Results

**Figure 1:**
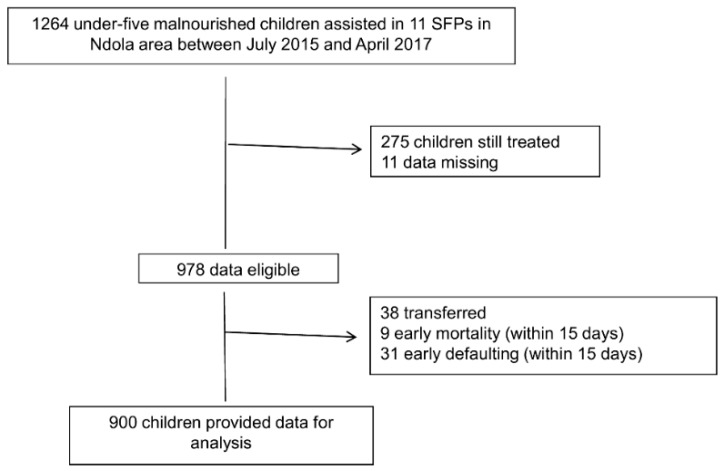
The study flowchart.

Data on 1264 malnourished Zambian children (6–59 months) who were seen between July 2015 and April 2017 were extracted from the database. Children still on rehabilitation at the moment of the study were excluded from the analysis. Formally transferred, early mortality, and early defaulting episodes (occurred within 2 weeks from admission) were not included. Eleven cases were excluded from the analysis because their records either had incomplete or missing baseline information. Therefore, the overall sample analyzed 900 children, all with accurate and complete information relevant to measuring the outcome of the intervention (Figure 1).

The main socio-demographic characteristics of the enrolled children in 2015–17, comorbidity and nutritional status at baseline, are reported in Table 1, and compared with data for 2012–14 previously published [20]. All children came from low socio-economic households. The youngest children (<18 months of age) were more likely to be affected by general acute malnutrition (OR 3.2, CI: 2.4–4.2), with more than 90% of children with kwashiorkor being less than 29 months of age. The high prevalence of kwashiorkor found at admission (20.4%) was consistent with the proportion of SAM cases presented in the annual Zambia country report [33].

### 3.1. First Results: Program Performance and Anthropometric Analysis

We evaluated the Rainbow 2015–17 outcomes and compared the results either with those of Rainbow 2012–14 and with the International Sphere Standards/UNHCR indicators, only available for SAM and MAM (Table 2).

The three main core performance outcomes (recovery, death, and defaulter rate) met the Sphere Standards, either for general or when SAM and MAM outcomes were split.

General improvements in all rates of these main indicators were noted when compared with Rainbow 2012–14, with a statistically significant increase in recovery rate for SAM (*p* = 0.01). Halving the mortality rates was the main goal achieved since in the first evaluation it was above the targets; for total: 5.6% in Rainbow 2012–14 vs. 3.1% in Rainbow 2015–17, *p* = 0.01; for SAM: 12.4% in Rainbow 2012–14 vs. 6.7% in Rainbow 2015–17, *p* = 0.006. The mean length of stay was above the UNHCR target, although for MAM the mean length of stay was exceeded the International standard only for one week (13.1 weeks ±7.4 SD). A longer period of recovery was needed by children with SAM (+4 weeks), with marasmatic children more likely to stay longer (OR 2.3, CI: 1.6–3.4), while children having kwashiorkor resolved edema earlier (OR 0.4, CI: 0.3–0.6). As compared to Rainbow 2012–14, a reduction in the mean length of stay was noted either for the total or for the two malnutrition groups (*p* < 0.001). 

Despite daily gains in mean grams per weight (2 g/kg/day ±1.5 SD), this measure was still below the international target of ≥3 g/kg/day for SFPs. Nevertheless, our findings are in line with reviews of the literature (between 1 and 2 g/kg/day) where programs supplemented with corn/soy blended flour (CSB) were evaluated [25,34]. We believe that sharing food within the household could have been a potential explanation for the quite poor mean weight gain since it is a common cultural practice, especially in food insecurity contexts [35]. Children admitted with SAM had higher weight gains then those with MAM, which might be due to simultaneous consumption of RUTF, even if erratic. A statistically significant increase in the average weight gain was noted when the outcomes were compared with that of Rainbow 2012–14 due to the new food schedule that allowed a reduction in the mean length of stay for recovered children, as proposed in the previous study [22].

As novel aspects of the community-based management of child malnutrition, Rainbow SFPs have been effective even in the nutritional rehabilitation of those children, although International guidelines do not provide specific targets for children who are underweight: 85.9% recovered; 13.8% defaulters; 0.3% deaths; average weight gain 1.7 g/kg/die ±1.3 SD; mean length of stay 20.3 weeks ±10 SD.

To identify the main predictors of mortality and defaulting, we performed univariate and multivariate (forward stepwise model) Cox proportional risk regression analyses (HR, 95% CI). The available characteristics at baseline and nutritional response to rehabilitation were analyzed separately (Table 3). Baseline characteristics included in the multivariate analysis were age below 18 months, socio-demographic conditions (rural area, orphan), HIV infection, anthropometric assessment (nutritional edema, WAZ <–3, ZMUAC <–2) and health problems at admission. Nutritional rehabilitation included gains in anthropometric parameters during the admission in the SFPs (WAZ gain, weight gain, ZMUAC gain).

The HIV infection still remained the major predictor of mortality (HR 5.5; CI: 1.9–15.9), together with SAM, defined as kwashiorkor (HR 3.5; CI: 1.2–9.5). Nutritional edema was associated with a high risk of mortality, and confirmed the importance of kwashiorkor as a public health problem, but was often not perceived as worrisome by guardians [33]. Severe underweight status at admission (WAZ<–3) also posed a greater risk of death (HR 4.6; CI: 1.3–16.1). This result was in line with the previous Rainbow study and highlighted the premise that the prevention and management of underweight status, considered a second priority criterion of admission, should be recognized as an essential part of the program against childhood malnutrition [23,24]. Compared to Rainbow 2012–14, predictors of mortality were no longer noted to be risk factors for defaulting. We identified that living in the rural area (HR 2.3; CI: 1.3–4.2) and being an orphan of any parent (HR 3.2; CI: 1.5–6.8) were the only two baseline variables associated with defaulting. Considering the nutritional response to treatment, the poor gain in any anthropometric parameters was independently associated either with case-fatality or defaulting. Specifically, when considering the multivariate Cox analysis, low weight gain and ZMUAC gain were more predictive of mortality, while poor WAZ gain and ZMUAC gain of defaulting.

**Figure 2:**
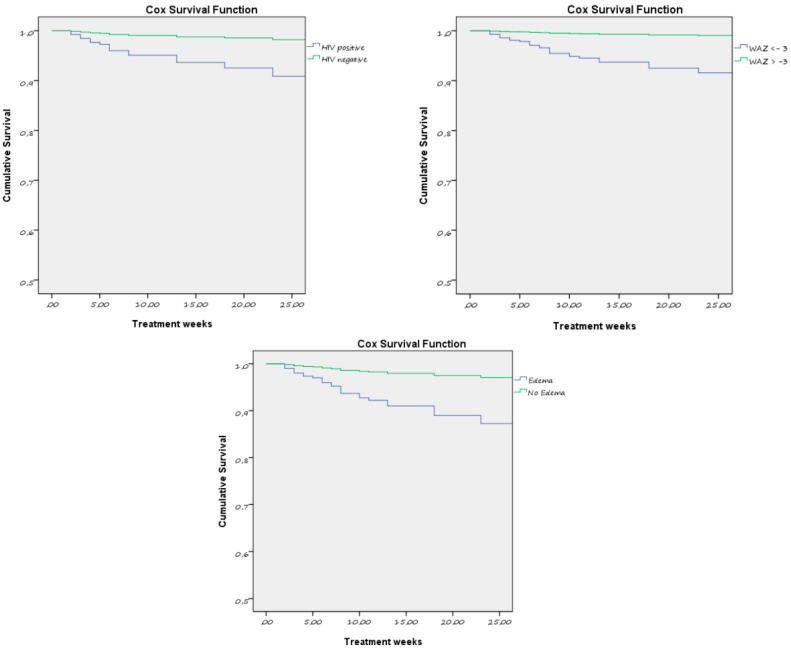
The Cox survival analysis. The outcome of death by HIV status, weight for age z-score, and kwashiorkor at baseline.

Figure 2 shows the result of the Cox survival analysis (per outcome death) by HIV status, baseline WAZ, and the presence of edema. Children affected by any of these critical conditions—defined as HIV infection, very low weight-for-age (WAZ <–3) and kwashiorkor (presence of nutritional edema)—were more likely to die than their counterparts.

### 3.2. Second Results: HIV Counseling and Testing

**Figure 3:**
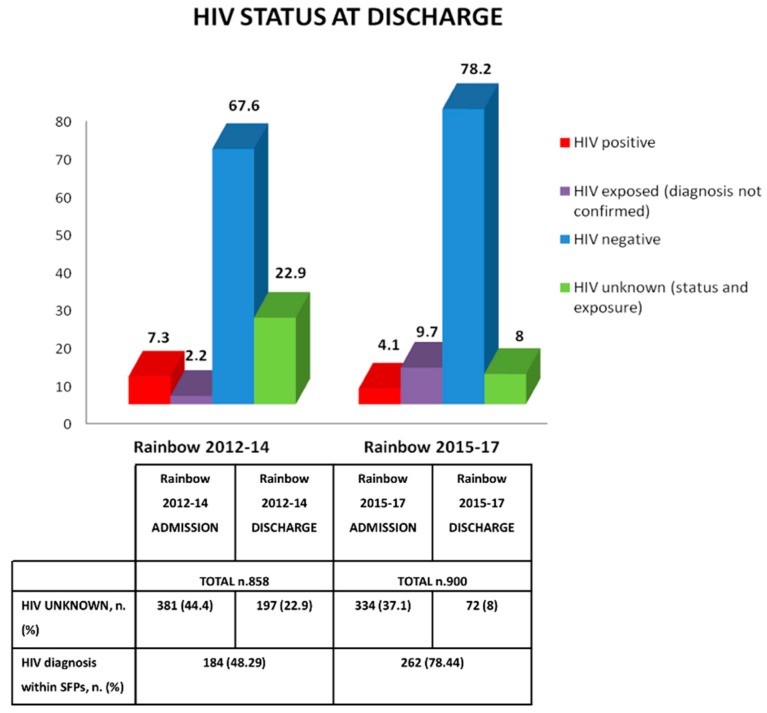
The HIV status at discharge: comparison between Rainbow 2012–14 and 2015–17.

Figure 3 presents the data on the HIV status of children at the time of discharge, comparing results with Rainbow 2012–14. The general number of children with an HIV infection decreased over the years (7.3% vs. 4.1%) and conversely, the number of HIV negative children increased (67.6% vs. 78.2%), reflecting the efforts of the Zambian government in promoting HIV PMTCT after the launch of new Guidelines on HIV. In fact, at the time of data collection, the country had adopted and progressively implemented WHO Option B+, recommending a lifelong triple-combination ART for all confirmed HIV-infected children regardless of the CD4 count and/or WHO clinical stage [36].

In addition, the most incisive goal on HIV reached by the Rainbow SFPs was seen in the increased access to HIV diagnosis during the enrollment in the programs. As compared to the nearly 49% of diagnoses made in Rainbow 2012–14, nearly 79% of new HIV diagnoses were made in Rainbow 2015–17 (30% greater; *p* < 0.001). Among the 83 children who were HIV exposed but still without an HIV diagnosis (9.7%), nearly 65% were less than 18 months of age, so presumably, definitive test results were not yet available.

## 4. Discussion

The Rainbow experience in Zambia supports the hypothesis that monitoring and evaluating activities improve the sustainability and effectiveness of community-based programs for child malnutrition that already exist and are well-implemented in the field [5,37,38,39]. Integrated community-based programs that promote general child malnutrition treatment and prevention, hold the potential to reduce the prevalence of acute malnutrition by reducing the incidence and enhancing treatment effectiveness [8,40,41]. Nutritional rehabilitation has not been considered a stand-alone intervention within nutritional programs: nutritional counseling, IYCF knowledge, health education, HIV/AIDS counseling, testing and diagnosis, immunization sensitization and awareness, control of infections and malaria, TB prevention and screening, must be integrated in CMAM protocols as these are critical primary care elements [42,43,44]. Providing this such innovative approach allows for the early optimization of nutritional status, with a positive impact on the prevention of acute malnutrition [23,24]. Recently, it has also been proven that combined protocol for MAM and SAM treatment with RUTF could be cost-effective [45]. Similarly, Rainbow’s innovative protocol, providing an integrated approach for the treatment and prevention of MAM, SAM and underweight, has shown to be effective, culturally acceptable, and affordable, while using local food plus fortified blended flour locally produced in an area with the erratic availability of RUTF. The efficacy of high energy supplements for child malnutrition management is already well documented in the literature [25,34,46,47,48].

As a novel study, the present analysis also highlighted that well-trained and supervised community volunteers are capable of identifying and managing cases of uncomplicated malnutrition, providing accurate, reliable, and trustable data [49]. These results are not easily demonstrable in community programs as discussed in field reports, where data collection and analyses are often not rigorously performed [5].

As first aim of the current study, outcomes of 2015–17 met the Sphere International Standards, either in general or when considering the different types of malnutrition separately, with statistically significant improvements compared to those of 2012–14. Improving the food schedule coupled with nutritional counseling [23,25,50] increased the average weight gain significantly and shortened the mean length of stay for recovered children, as proposed in the previous Rainbow study. Halving mortality rates was the main observable goal, in general, or when considering MAM and SAM separately. Our findings show outcomes similar to other studies evaluating community-based programs for moderate malnutrition despite, in some cases, a lower weight gain [51,52], or a better cure rate but higher mortality rates [53]. It was difficult to compare the length of stay with results from other field studies since “non-cured/non-responder” was not an outcome of Rainbow SFPs as nutritional assistance was ensured to all children until recovery occurred. 

When investigating the factors contributing to negative outcomes, the Cox proportional risk analysis showed that very low weight-for-age at baseline (<–3SD), kwashiorkor and, in particular, HIV infection were the main predictors of mortality. Along with the survival risk, it is widely recognized that children affected by HIV/AIDS are at serious risk of developmental delays, which is also reported for those exposed to HIV in utero but are born uninfected, and for those whose parents are affected by HIV [54,55]. More research is needed to investigate these results within the specific Zambian context. The poor gain in anthropometric parameters (WAZ gain, weight gain, and ZMUAC gain) during nutritional rehabilitation was independently associated either with case-fatality or defaulting. This result was in line with the previous Rainbow study and highlighted the premise that the prevention and management of underweight status, considered a second priority criterion of admission, should be recognized as an essential part of the program against childhood malnutrition [23,24]. Future research on growth velocity should be performed in order to define better indices for predictors of child mortality [56].

As a second aim of this study, a significant reduction in children with an unknown HIV status at the time of discharge (nearly 35% less) was noted, reflecting the large effort put into VCT for HIV/AIDS for which Rainbow’s community volunteers were strongly trained. We underline the great importance of this result since this improvement was reached with no additional costs for the program besides routine capacity building activities (training and refresher courses). Moreover, the decreased number of children who were HIV infected over the years was in line with the country’s report which stated a decline in HIV MTCT from 14.9% in 2013 to less than 9% in 2014 [36,57]. The efforts of the Zambian government in promoting HIV PMTCT was also supported by the increased number of HIV infected children receiving antiretroviral treatment, 70% in 2015–17 compared to the 52% in 2012–14. Since it is well known that the HIV infection is the most important predictor of mortality among HIV infected, especially when not treated or timely tested [58,59], we can presume that the greatest efforts in VCT and knowledge in HIV/AIDS, both at the health district and at the community level, might have contributed in lowering mortality rates within the Rainbow SFPs.

### Limitations of the Study

For ethical and humanitarian reasons, the same protocol and treatment were ensured to all the children admitted in Rainbow SFP, so that in the absence of a control group we can only estimate that improvements in mortality outcomes are due to the changes made to the protocol.

In regards to RUTF consumption, unfortunately, no specific evaluations were done due to the lack of consistent information on RUTF taken and/or disruption of OTP services. We can presume that a simultaneous consumption of RUTF by children with SAM, even if at times erratic, might have contributed to their weight gain. Full collaboration and dialogue among different local stakeholders dealing with malnutrition should be therefore promoted and constantly enhanced.

Finally, we emphasize that there is still a lack of common standard protocols for evaluating the effectiveness of community-based programs delivered in non-emergency contexts, with Sphere indicators still being the main markers for the assessment of CMAM performance. Conditions for delivering CMAM programs are not the same in non-emergency and emergency contexts, and for this reason, relying only on Sphere guidelines to measure program performance may not be comprehensive enough [6]. More research is needed to identify other field-based indicators which should be included in the evaluation of the effectiveness of CMAM, such as specific indicators for underweight and relapse malnutrition rates. In fact, we have identified a high proportion of relapses that need further investigation, in accordance to the recent literature [60].

## 5. Conclusions

The Rainbow SFPs provide a sustainable community-based approach for the integrated treatment and prevention of malnutrition in Zambia that is effective in reduction of mortality among under-5 malnourished children, whether MAM, SAM or underweight. Moreover, Rainbow SFPs act as efficient entry points for child growth surveillance, nutrition and health promotion, facilitating access to HIV/TB treatment and care for Zambian children.

The consistent monitoring and evaluation of the programs with the identification of critical areas and adjustments made accordingly, as well as the continuous technical assistance and support, specific trainings, and refresher courses of the community volunteers, resulted in significant gains in the process and generated positive outcomes, with the consequent enhancement of the program’s performance.

When CMAM is not fully implemented or scaled-up, our results emphasize the need for nutritional programs already implemented in the field to identify coping strategies for not neglecting any form of child malnutrition, especially most severe cases. Key to the process is a holistic and multi-sectoral approach that facilitates the integration between nutrition and health, taking into account the local environment, affordable resources, and culturally accepted actions.

## Figures and Tables

**Table 1:** The socio-demographic, health and nutritional characteristics of children at the baseline, Rainbow 2015–17 and 2012–14.

Variables	Value 2015–17	Value 2012–14
Male, n. (%)	439 (48.8)	428 (49.9)
Age in months, mean ± SD	19.7 ± 9.9	19 ± 9.4
<18 months of age	452 (50.2)	455 (53)
Rural area, n. (%)	139 (15.4)	163 (19)
Parental status, n. (%)		
Orphans of one parent	32 (3.6)	33 (3.9)
Orphans of both parents	15 (1.7)	15 (1.7)
Single guardian	238 (26.4)	NA
Disability, n. (%)	23 (2.6)	NA
Twin, n. (%)	42 (4.7)	62 (7.2)
Caregiver’s age, mean ± SD [min–max]	28.4 ± 9.2	29 ± 10
	[15–69]	[13–78]
Referred from, n. (%)		
Hospital	23 (2.6)	46 (5.4)
Local health facility	186 (20.7)	279 (32.5)
Community	691 (76.7)	533 (62.1)
HIV status, n. (%)		
Infected	25 (2.8)	51 (5.9)
Uninfected	541 (60.1)	426 (49.7)
Status unknown	334 (37.1)	381 (44.4)
Non-breastfed children, n. (%)	564 (62.7)	NA
Months of age, mean ± SD	14.9 ± 6.9	
Admission criteria: MAM, n. (%)	242 (26.9)	323 (37.6)
<18 months of age	145 (59.9)	195 (60.4)
Admission criteria: SAM, n. (%)	346 (38.4)	241 (28.1)
<18 months of age	207 (59.8)	146 (60.6)
Admission criteria: Underweight, n. (%)	312 (34.7)	294 (34.3)
<18 months of age	100 (32.1)	114 (38.8)
Presence of edema, n. (%)	184 (20.4)	77 (9)
Relapses of malnutrition event, n. (%)	129 (14.3)	68 (7.9)
Weight (kg), mean ± SD	7.6 ± 1.5	7.8 ± 1.6
WAZ, mean ± SD	−3.1 ± 0.9	−2.8 ± 1.1
MUAC (cm), mean ± SD	12.2 ± 1	12.4 ± 1
ZMUAC, mean ± SD	−2.4 ± 1	NA
Health problems, n. (%)	429 (47.7)	299 (34.9)
Fever	75 (8.3)	73 (8.5)
Diarrhea	118 (13.1)	71 (8.3)
Lack of appetite	124 (13.8)	66 (7.7)
Cough/Sneezing	99 (11)	28 (3.3)
Malaria	4 (0.4)	7 (0.8)
Others	9 (1.1)	54 (6.3)

MAM = moderate acute malnutrition; MUAC = mid-upper arm circumference; SAM = severe acute malnutrition; WAZ = weight for age Z-score; ZMUAC = MUAC for age Z-score.

**Table 2:** The Rainbow 2012–14 vs. 2015–17 outcomes, and the International Standards.

	Total			MAM			SAM			International Standards (Sphere Project/UNHCR) [23,31]	
Indicators	Rainbow 2012–14 (n.858)	Rainbow 2015–17 (n.900)	*p-*Value	Rainbow 2012–14 (n.323)	Rainbow 2015–17 (n.242)	*p-*Value	Rainbow 2012–14 (n.241)	Rainbow 2015–17 (n.346)	*p-*Value	Acceptable	Alarming
**Recovered, n. (%)**	709 (82.6)	771 (85.7)	0.08	278 (86.1)	219 (90.5)	0.1	177 (73.5)	284 (82.1)	*0.01*	>75%	<50%
**Defaulters, n. (%)**	101 (11.8)	101 (11.2)	0.72	36 (11.1)	17 (7)	0.09	34 (14.1)	41 (11.8)	0.42	<15%	>30%
**Deaths, n. (%)**	48 (5.6)	28 (3.1)	*0.01*	9 (2.8)	6 (2.5)	0.8	30 (12.4)	21 (6.1)	*0.006*	<3% for SFPs	>10%
										<10% for TFPs	
**Mean length of stay, weeks (SD)**	19.3 ± 11.5	16.6 ± 9.6	*<0.001*	19.3 ± 11.9	13.1 ± 7.4	*<0.001*	22 ± 11.8	15.9 ± 9.6	*<0.001*	<12 weeks for SFPs	
										<3–4 weeks for TFPs (IC till full recovery)	
										<60 days for TFPs (IC and OTPs combined)	
**Average weight gain, g/kg/day (SD)**	1.7 ± 1.2	2 ± 1.5	*<0.001*	1.7 ± 1	1.9 ± 1.2	*0.03*	2 ± 1.3	2.4 ± 1.7	*0.007*	≥3 g/kg/day for SFPs	
										≥8 g/kg/day for TFPs (IC till full recovery)	
										≥4 g/kg/day for TFPs (IC and OTPs combined)	

IC = inpatient care; MAM = moderate acute malnutrition; OTPs = outpatient therapeutic programs; SAM = severe acute malnutrition; SFPs = supplementary feeding programs; TFPs = Therapeutic feeding programs.

**Table 3:** The predictors of mortality and defaulting. Cox proportion risk analysis.

		Predictors of Mortality						Predictors of Defaulting					
		Univariate Analysis			Multivariate Analysis			Univariate Analysis			Multivariate Analysis		
		HR	95% CI	*p-*Value	HR Exp (B)	95% CI	*p-*Value	HR	95% CI	*p-*Value	HR Exp (B)	95% CI	*p-*Value
**Baseline characteristics**	**Age < 18 months**	1.04	0.49–2.19	0.917	-	-	-	0.83	0.56–1.23	0.350	-	-	-
	**Rural Area**	0.24	0.03–1.78	0.164	-	-	-	2.48	1.61–3.82	*<0.001*	2.31	1.27–4.21	*0.006*
	**Orphan**	1.69	0.40–7.14	0.473	-	-	-	2.41	1.26–4.64	*0.008*	3.22	1.52–6.81	*0.002*
	**HIV infection**	8.11	2.85–23.04	*<0.001*	5.53	1.92–15.94	*0.002*	1.02	0.32–3.27	0.968	-	-	-
	**WAZ <–3**	9.37	2.83–31.06	*<0.001*	4.57	1.30–16.11	*0.018*	1.01	0.51–2.00	0.983	-	-	-
	**ZMUAC <–2**	8.11	1.92–34.20	*0.004*	-	-	-	1.50	0.99–2.30	0.062	-	-	-
	**Kwashiorkor/edema**	4.59	2.14–9.85	*<0.001*	3.51	1.29–9.51	*0.014*	1.10	0.65–1.86	0.724	-	-	-
	**Health problems**	1.75	0.82–3.73	0.149	-	-	*-*	1.34	0.91–1.99	0.140	-	-	-
**Nutritional response characteristics**	**Weight gain (g/kg/die)**	0.42	0.34–0.51	*<0.001*	0.59	0.48–0.74	*<0.001*	0.57	0.49–0.65	*<0.001*	-	-	*-*
	**WAZ gain**	0.17	0.12–0.24	*<0.001*	-	-	-	0.29	0.23–0.38	*<0.001*	0.58	0.41–0.82	*0.002*
	**ZMUAC gain**	0.21	0.16–0.28	*<0.001*	0.35	0.24–0.51	*<0.001*	0.32	0.26–0.40	*<0.001*	0.44	0.33–0.59	*<0.001*

CI = confidence intervals; HR: hazard ratio; WAZ = weight for age Z-score; ZMUAC = MUAC for age Z-score.

## Abbreviations

AIDS	Acquired Immune Deficiency Syndrome
ART	Antiretroviral Therapy
ARV	Antiretroviral
CBOs	Community-based Organizations
CI	Confidence Interval
CMAM	Community-Management of Acute Malnutrition
CSB	Corn soya blended food
CTC	Community-based Therapeutic Care
DHMTs	District Health Management Teams
HAART	Highly active antiretroviral therapy
HEPS	High energy protein supplement
HIV	Human Immunodeficiency Virus
HR	Hazard Ratio
IC	Inpatient Care
IMAM	Integrated Management of Acute Malnutrition
IYCF	Infant and Young Child Feeding
MAM	Moderate Acute Malnutrition
MUAC	Mid-Upper Arm Circumference
NGOs	Non-Governmental Organizations
OR	Odds Ratio
OTP	Outpatient Therapeutic Care
PMTCT	Prevention Mother-to-Child Transmission
RUTF	Ready-to-use Therapeutic Food
SAM	Severe Acute Malnutrition
SD	Standards Deviation
SFP	Supplementary Feeding Programs
SPSS	Statistical packages for social sciences
TFP	Therapeutic Feeding Programs
TB	Tuberculosis
TDRC	Tropical Diseases Research Centre
UNHCR	United Nations High Commissioner for Refugees
UNICEF	United Nations International Children’s Emergency Fund
VCT	Voluntary counseling and testing
WAZ	Weight-for-Age Z-score
WHO World Health Organization	World Health Organization
WHZ	Weight-for-Height Z-score
WLZ	Weight-for-Length Z-score
ZMUAC	Mid-Upper Arm Circumference for Age Z-score
